# The Influence of Plant Stress Hormones and Biotic Elicitors on Cyclotide Production in *Viola uliginosa* Cell Suspension Cultures

**DOI:** 10.3390/plants11141876

**Published:** 2022-07-19

**Authors:** Blazej Slazak, Aleksandra Jędrzejska, Bogna Badyra, Reza Shariatgorji, Anna Nilsson, Per E. Andrén, Ulf Göransson

**Affiliations:** 1 W. Szafer Institute of Botany of the Polish Academy of Sciences, 46 Lubicz, 31-512 Krakow, Poland; ajedrzejska@wp.pl; 2 Pharmacognosy, Department of Pharmaceutical Biosciences, Uppsala University, P.O. Box 574, 751 23 Uppsala, Sweden; ulf.goransson@farmbio.uu.se; 3 Laboratory of Neurobiology, Nencki-EMBL Center of Excellence for Neural Plasticity and Brain Disorders: BRAINCITY, Nencki Institute of Experimental Biology of the Polish Academy of Sciences, 02-093 Warsaw, Poland; bogna.badyra@gmail.com; 4 Spatial Mass Spectrometry, Science for Life Laboratory, Department of Pharmaceutical Biosciences, Uppsala University, P.O. Box 591, 751 24 Uppsala, Sweden; reza.shariatgorji@farmbio.uu.se (R.S.); anna.nilsson@farmbio.uu.se (A.N.); per.andren@farmbio.uu.se (P.E.A.)

**Keywords:** plant in vitro cultures, cyclic peptides, host-defense peptides (HDPs), medicinal peptides, plant stress response, MALDI-MS

## Abstract

Cyclotides are macrocycle peptides produced by plants from several families, including Violaceae. These compounds have the potential for applications in medicine, bioengineering and crop protection thanks to their multiple biological activities. In most cases, cyclotides are extracted from plant material. Plant cell culture provides a viable and sustainable form of plant biomass production Cyclotides are host defense peptides. The aim of the current study was to test whether different plant stress hormones and biological elicitors have effects on cyclotide production in *Viola uliginosa* suspension cultures. Different concentrations of jasmonic acid (JA), salicylic acid (SA), abscisic acid (ABA) and neutralized pathogens were tested. The cyclotide production was assessed using MALDI-MS. Five major peptides produced by *V. uliginosa* cultures were chosen for analysis, of which one was sequenced de novo. The treatments had little influence on the suspension’s growth, with the exception of 100 μM SA, which enhanced the biomass increase, and 100 μM ABA, which was toxic. Significant increases in the production of three cyclotides (viul M, cyO13 and cyO3) were observed in suspensions primed with JA (50 μM, 100 μM, 200 μM) after 14 days of culturing. Biotic elicitors had no observable effect on cyclotide production. The current study indicates that some cyclotides in *V. uliginosa* are triggered in response to JA. The stress plant hormones can be used to enhance plant cell culture-based production systems.

## 1. Introduction

Cyclotides are peptides produced by plants of several families: the Violaceae, Rubiaceae, Cucurbitaceae, Fabaceae, Solanaceae and Poaceae [1,2,3,4,5,6,7]. Cyclotides are characterized by around 28–36 amino-acid head-to-tail cyclic peptide backbones and three disulfide bonds in knotted conformation [8,9]. Cyclotides are attractive for bioengineering and medicine due to their molecular stability and inherent biological activities, including uterotonic, hemolytic, anti-HIV, cytotoxic, and antimicrobial action [10,11,12,13,14,15].

Because of the complex molecular structure of cyclotides, their synthesis can be challenging and expensive. In many cases, it is still more feasible to extract the peptides from plant material [16,17]. However, extraction requires large quantities of plant material, and its availability may be a major issue, especially for rare plants producing unique cyclotides. In vitro culture methods for plants have been developed for several cyclotide-producing species: *Oldenlandia affinis*, *Viola odorata* and *V. uliginosa*. Such techniques offer high biomass production, sustainability and cyclotide yields higher than those from the crude plant material [18,19,20]. Plant cell suspension-based cyclotide production systems can be improved in relation to yield by modifying the culture conditions and applying different growth regulators or biological elicitors [16,19,21]. In addition, in vitro cultures are maintained in defined and controlled conditions, which provide a perfect system to investigate biological processes like the influence of stress factors on cyclotide production.

The presumed biological role of cyclotides is plant host defense [12,22]. However, it is unclear whether they are part of constitutive defenses or if their production is induced in response to biotic or abiotic factors. A study on *O. affinis* reported no response to external stimuli in terms of the gene expression levels of several cyclotides [23]. On the other hand, it has been shown in several studies that cyclotide production is dependent on external factors, environment or season [24,25,26]. Moreover, we have shown recently that some peptides in *V. odorata* are produced in response to spider mite infestation [27].

The current study describes the influence of stress hormones and biological elicitors on cyclotide production in *V. uliginosa* cell suspension cultures.

## 2. Results

### 2.1. The Influence of Plant Stress Hormones and Elicitors on V. uliginosa Suspension Culture Growth

**Figure 1:**
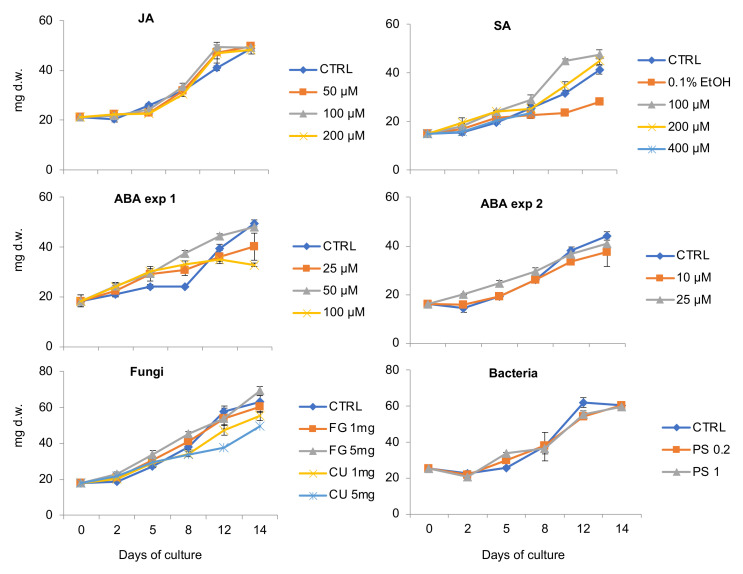
Growth curves for *V. uliginosa* cell suspension cultures treated with plant stress hormones (JA, SA, ABA) and the biotic elicitors, neutralized fungi (FG—*Fusarium graminearum* and CU—*Colletotrichum utrechtse*) and bacteria (PS—*Pseudomonas syringae*)–in different concentrations. All treatments were compared with the untreated controls (CTRL).

Most of the tested plant stress hormones had little effect on the suspension culture growth (Figure 1). Faster growth than the controls was observed after treatment with 100 μM SA. In this experiment, 0.1% ethanol showed signs of toxicity and negatively influenced the culture growth. Similarly, 100 μM ABA as well as CU neutralized fungal biological elicitor had adverse effects on the cultures (Figure 1). The color of the cultures changed from yellow to brown under the treatment of JA and ABA stress hormones (not shown).

### 2.2. Major Cyclotides Produced by V. uliginosa Cell Suspensions and Discovery of a Novel Cyclotide

**Figure 2:**
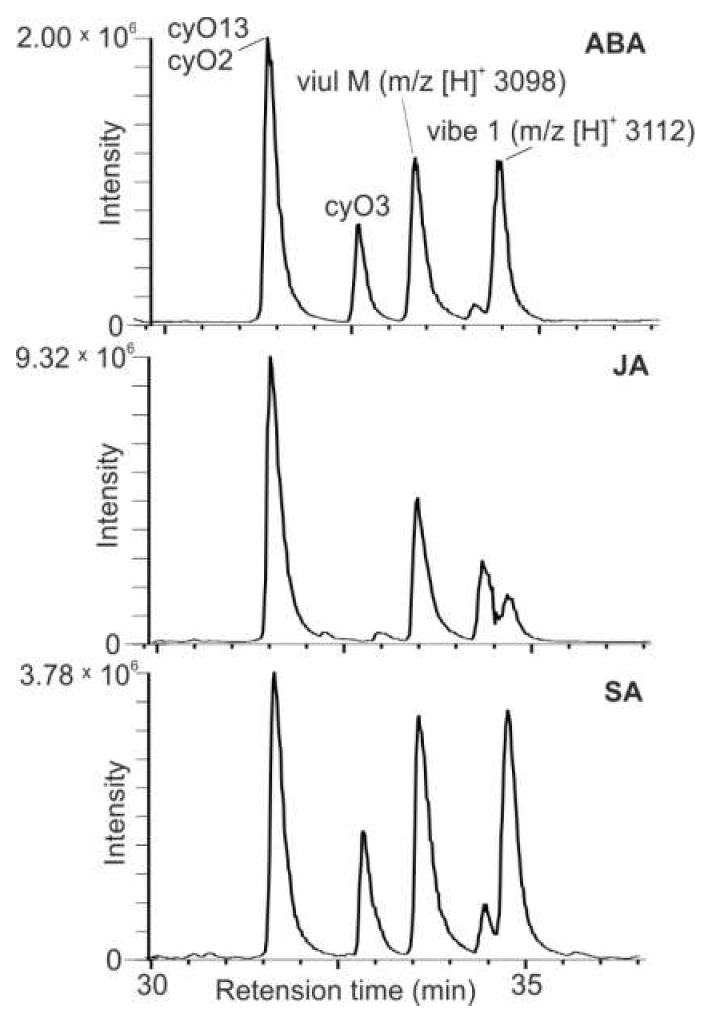
LC-MS trace of an example extract made from cultures treated with SA, JA and ABA. The peaks corresponding to the five main constituents (*m*/*z* [m+H]^+^ 3098, *m*/*z* [m+H]^+^ 3112, cyO2, cyO3, cyO13) are marked.

To determine the main cyclotides produced by *V. uliginosa* cell suspensions, the extract was subjected to LC-MS analysis. The main peaks corresponded to five peptides: *m*/*z* [m+H]^+^ 3098, *m*/*z* [m+H]^+^ 3112, cyO2, cyO3, cyO13 (Figure 2). The three latter cyclotides were identified based on previous work [19].

**Figure 3:**
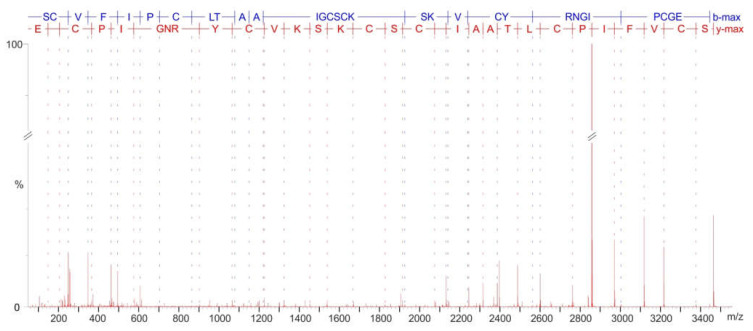
Peptide sequencing of the novel cyclotide (named viul M) produced in *V. uliginosa* cell suspension culture based on its MS/MS fragmentation spectrum. The isolated cyclotide was alkylated (carbamidomethylated cysteines) and digested with Glu-C endoprotease, resulting in a linear peptide subsequently analyzed with LC-MS/MS.

The ion *m*/*z* [m+H]^+^ 3098 corresponding to a cyclotide was noted to be present and denoted with its monoisotopic molecular weight (3097 Da) in the extracts from *V. uliginosa* suspension cultures before [19]. However, this peptide has never been identified and sequenced. It was purified from a larger scale extract and subjected to LC-MS/MS analysis after alkylation and enzymatic digestion. The peptide amino acid sequence was determined on the basis of the MS/MS fragmentation spectrum of the linearized peptide (after Glu-C cleavage, Figure 3) and the peptide fragment resulting from trypsin cleavage (Figure S1). The final sequence was found to be the following: GIPCGESCVFIPCLTAAIGCSCKSKVCYRN.

The MS/MS sequencing after alkylation and Glu-C cleavage allowed *m*/*z* [m+H]+ 3112 cyclotide to be identified as the previously described peptide, vibe 1 (Figure S2).

### 2.3. The Influence of Plant Stress Hormones and Elicitors on Relative Cyclotide Production in V. uliginosa Cell Cultures

**Figure 4:**
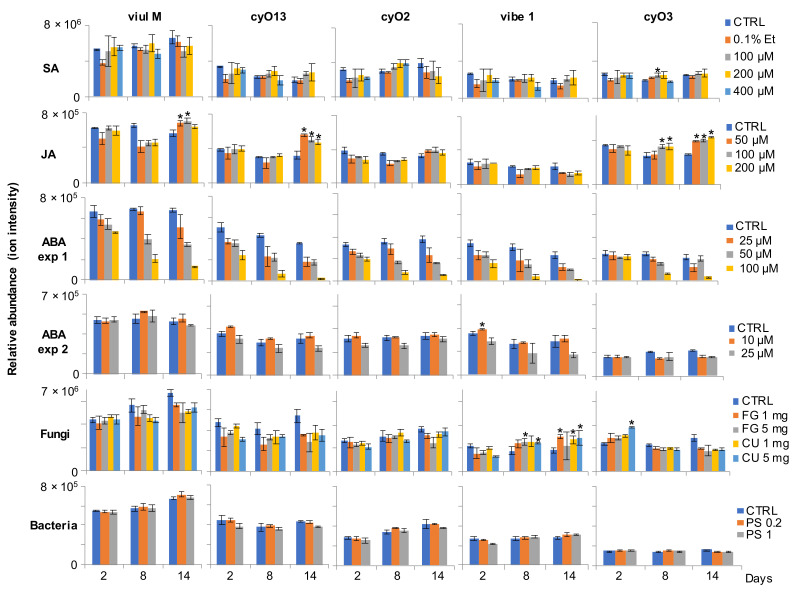
The influence of plant stress hormones (JA, SA, ABA) and biotic elicitors (neutralized fungi and bacteria) on the relative quantity of the five most abundant cyclotides (viul M, cyO13, cyO2, vibe 1, cyO3) produced by the *V. uliginosa* cell suspension cultures at different timepoints from treatment. Significant increases in cyclotide production in comparison to controls (ANOVA with *t*-test, *p* < 0.05) are marked with an asterisk. Each treatment and controls were in triplicates (n = 3).

Treatment with SA, in the majority of cases, did not cause significant changes in the production of the five assessed peptides in comparison to untreated controls. Significant differences (ANOVA with *t*-test, *p* < 0.05) were detected only in the case of cyO3 in eight-day-old cultures treated with 100 μM SA. Treatment with 0.1% ethanol appeared to have small detrimental effects on cyclotide production (Figure 4). The suspensions primed with JA produced significantly more viul M, cyO13 and cyO3 compared to controls after 14 days. In the case of cyO3, significant differences were also observed in cultures after eight days of treatment (Figure 4). The higher concentration of ABA that was used in ABA experiment 1 had toxic effects, as indicated by the low cyclotide content compared to the controls. The low ABA concentrations had little effect on cyclotide production (Figure 4). Of the biotic elicitors, the neutralized fungi (FG, CU) had effects on the production of vibe 1 cyclotide after eight and fourteen days of treatment. Treatment with neutralized bacteria had no effects on cyclotide abundance (Figure 4).

## 3. Discussion

The plant stress hormones and biological elicitors had only small effects on the growth rates of the *V. uliginosa* suspension cultures. In earlier studies on *V. odorata* and another cyclotide-producing plant, *O. affinis*, it was shown that other factors influence growth to a greater extent [20,28,29]. The highest variations in growth rate were observed in cultures derived from different explants or initiated using different combinations of plant growth regulators [20]. The growth rate can also be influenced to some extent by manipulating basic nutrients in the medium and adjusting light intensity [28]. However, in the current study, the toxicity of the stress hormones or elicitors was demonstrated only in cultures treated with higher concentrations of ABA.

The qualitative composition of the cyclotide extracts in *V. uliginosa* cultures in the current work did not differ from those previously reported. In both the current and earlier studies the most abundant cyclotides were *m*/*z* [m+H]^+^ 3098, *m*/*z* [m+H]^+^ 3112, cyO2, cyO3, cyO13 [19,30]. It appears that the production of the most abundant cyclotides in a given plant is regulated by plant growth regulators and is specific to a particular type of tissue. The composition of cyclotides has been shown to change greatly in *V. uliginosa*, and *V. odorata* cultured in vitro depending on the exogenous growth regulators added to the media and the type of callus tissue [18,19,29]. In *O. affinis*, cultures grown on media supplemented with 0.4 mgL^−1^ 2,4-D exhibited the fastest growth. However, these suspensions did not synthetize kB1, the most prominent cyclotide found in different organs of the plant and the explant (leaf) from which they were derived [20].

**Figure 5:**
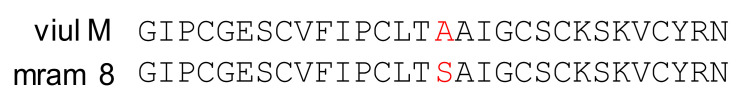
The alignment of the novel cyclotide viul M isolated from *V. uliginosa* suspension cultures with mram 8 identified earlier [19]. The difference in sequence is marked in red.

Two cyclotides produced by *V. uliginosa* suspension cultures were identified in the present study. The peptide with *m*/*z* [m+H]^+^ 3112 was found to be vibe 1, a cyclotide described previously from *V. betonicifolia,* a violet from Sri Lanka [31]. It is not surprising that similar peptides are found in different species. In fact, many species from the Violaceae share similar cyclotide patterns [4]. In some cases, the same cyclotides are found in non-related species from different plant families; for example, kB1, which is found in species from the Rubiaceae and Violaceae [32,33]. Another cyclotide–*m*/*z* [m+H]^+^ 3098–was newly sequenced in the present study and named viul M. A search for viul M in Cybase [34] (http://www.cybase.org.au/ accessed on 4 February 2022) and Uniprot (https://www.uniprot.org/, accessed on 4 February 2022) yielded no matching sequences. This indicates that viul M is a novel cyclotide. The amino acid sequence of viul M is very similar to mram 8, described from *V. uliginosa* cultures in a previous work, with only one substitution (Figure 5) [19].

The current study indicated that the best factor in terms of enhancing cyclotide production in *V. uliginosa* suspensions was JA. The cultures primed with JA showed a significant increase in abundance of three out of the five assessed cyclotides. Very little is known about the influence of stress factors on cyclotide production. It was shown by Dörnenburg (2010) that treatment of *O. affinis* suspensions with a biological elicitor—chitosan—substantially increased kB1 production [16]. The results of the present study indicate that cyclotide production can be enhanced with stress hormones. It has been shown in multiple studies that stress hormones like SA and jasmonates (including JA) elicit the biosynthesis of biologically active secondary metabolites [35]. For example, methyl jasmonate has been found to enhance production of flavonoids in *Hypericum perforatum* suspension cultures [36]. The present study assessed the production of cyclotides after different times of culturing, which allowed us to pinpoint when the highest yields can be expected. In the case of JA, the significant differences compared to untreated controls were observed after 14 days of culturing, at the peak of the log growth phase. It appears that the effects of JA build over time. It is also possible that JA influences the dynamics of biosynthesis and decomposition processes of certain peptides, leading to higher cyclotide content later in the culturing time. In an earlier study on the same *V. uliginosa* suspension cultures we showed that peptides differ in turnover rates and that regulation of these processes may have a large impact on shaping cyclotide production patterns [30]. SA and ABA hormones had smaller effects, thus linking the regulation of cyclotide production specifically to the JA pathway. On the other hand, the influence of other plant growth regulators present in the experiment may, to some extent, suppress the effects. In vitro cultures are valuable for studying how different stimuli influence plant cell biology because the experimental factors can be strictly controlled. However, when discussing the results, one should always be aware that the cells are not in their normal physiological state when in suspension. In particular, to sustain cell division, endogenous growth regulators need to be added to suspensions.

The current study indicates that some cyclotide production can be regulated via the jasmonic acid signaling pathway. JA and other jasmonates are well-known plant stress signaling molecules that trigger and regulate plant responses to mechanical damage or insect herbivory [37]. The results of the present study seem to corroborate the results of our recent work investigating the interaction between violets and spider mites. We showed that the production of certain peptides in *V. odorata* is triggered in response to spider mite infestation [27]. However, an earlier work investigating changes in cyclotide gene expression in *O. affinis* indicated that, in this plant, the peptides are produced as an innate defense [23]. It has to be noted that violets and *O. affinis* belong to different, non-related plant families (Violaceae and Rubiaceae). The cyclotide genes and the structures of the precursor proteins they encode are, in many aspects, different in these two species [38]. This is possibly associated with distinct biological roles and regulation of the production of cyclotides in these plants. It seems that in one species, some cyclotides are produced constitutively and in the other species, the peptides can be regulated in response to stress.

In conclusion, the current study demonstrates the influence of plant stress hormones on cyclotide production in *V. uliginosa* suspensions. A significant elevation of three out of five investigated cyclotides compared to non-treated controls was recorded in suspensions treated with JA after 14 days of culturing. Two new cyclotides were described from the *V. uliginosa* suspensions—one new for the species (vibe 1) and one novel (viul M).

## 4. Materials and Methods

### 4.1. V. uliginosa Cell Suspension Initiation and Culture Conditions

*Viola uliginosa* Besser plants were obtained from the collection of Professor Elżbieta Kuta (Cracow-Ugorek, Poland). The cell suspensions were initiated in the same way as previously described [19]. The callus tissue used to initiate the cell suspensions was derived from leaf fragments grown on Murashige and Skoog (MS) [39] basal medium supplemented with 2 mgL^−1^ 2,4-dichlorophenoxyacetic acid (2,4-D), 2 mgL^−1^ kinetin (KIN) and 30 gL^−1^ sucrose (2 + 2 media), solidified with agar. The callus tissue was transferred to 250 mL Erlenmeyer flasks with liquid media of the same composition, placed on a rotary shaker and cultured for 3–4 weeks. Subsequently, cell suspensions were obtained by draining the callus tissue and larger aggregates through a sieve. Sub-cultures were prepared every two weeks by transferring 25 mL of suspension into 75 mL of fresh media in 250 mL Erlenmeyer flasks. All plant growth regulators and MS media were supplied by Sigma-Aldrich, St. Louis, MO, USA.

All cultures were maintained on a rotary shaker in a culture chamber under a day–night temperature of 20 ± 2 °C and a photoperiod of 16 h/8 h with a light intensity of 60–90 μmoL m^−2^s^−1^.

### 4.2. Suspension Culture Treatments and Sample Preparation

Each treatment was tested on a different occasion in a separate experiment with its own untreated controls. Each treatment and control were prepared and analyzed in triplicate in three separate flasks. We used 50 mM stocks of three plant stress hormones, which were prepared in ethanol for jasmonic acid (JA) or salicylic acid (SA) and 1M NaOH for abscisic acid (ABA). At the same time, the treatments with biotic elicitors were prepared. *Fusarium graminearum* (FG) and *Colleotrichum utrechtense* (CU) plant pathogen fungi obtained from a previous study [22] were grown for five days in potato dextrose broth (PDB) media (Sigma). Subsequently, the mycelia were filtered out, autoclaved, and freeze-dried. *Pseudomonas syringae* (PS) bacteria were grown in LB media (Sigma) then the cultures were neutralized by autoclaving. Each stress factor or biotic elicitor was added on day 0 of each experiment. Each experiment was prepared by adding 15 mL of two-week-old culture to 35 mL of fresh 2+2 media with an appropriate amount of stock hormone solution or biotic elicitor to reach the desired final concentration in 100 mL flasks. The following stress hormone concentrations were tested: JA–50 μM, 100 μM, 200 μM; SA–100 μM, 200 μM, 400 μM. ABA was tested in two separate experiments (exp. 1, exp. 2) in concentrations: 25 μM, 50 μM, 100 μM (exp. 1) and 10 μM, 25 μM (exp. 2). To assess the influence of alcohol added with the stock hormone solutions, the SA experiment included an additional control treated with 0.1% ethanol. The fungi were tested by adding 1 mg or 5 mg of dry mycelia to every 100 mL of culture (0.5 mg and 2.5 mg per 50 mL). To test the bacteria, either 0.2 mL or 1 mL of the neutralized bacteria was added per 100 mL of culture.

The 1 mL samples were drawn at days 0, 2, 5, 8, 12 and 14. The sampled suspensions were filtered through previously oven-dried and weighed filter paper. The filters with the cultures were freeze-dried and weighed, and the weight of the biomass was determined by deducting the weight of the filter paper. Finally, the dry material (1–5 mg) was scraped from the filter, weighed, and placed in separate Eppendorf tubes for extraction.

### 4.3. Relative Quantitation of Cyclotides

The samples were prepared and analyzed according to previously published protocols [24,27]. All the freeze-dried samples, 2–4 mg of dry weight (d.w.), were pulverized and extracted with 200 µL of aqueous 30% acetonitrile (ACN), 0.05% trifluoro acetic acid TFA per mg of d.w., using TissueLyser (Qiagen, Germantown, MD). Extracts were centrifuged, and the supernatant was collected. Preliminary experiments with serial dilutions of extracts were performed to assess the maximum detection point of the MALDI-MS method. The extracts from several samples were also analyzed with LC-MS using UPLC-QToF nanospray MS (Waters nanoAcquity, 75 µm × 250 mm 1.7 µm BEH130 C18 column, Waters QToF Xevo) to determine the main cyclotide constituents. Finally, all the samples were diluted fivefold prior to MALDI-MS semi-quantitative analysis in order to place the intensity values for selected cyclotide ions in a linear range. Metal plates spotted with 0.5 µL of extracts were sprayed with 2,5-dihydroxybenzoic acid (35 mg/mL in 50% ACN and 0.2% TFA) matrix in six passes at 95 °C using an automatic TM-sprayer (HTX-Technologies LLC, Chapel Hill, NC, USA) with the following parameters: nitrogen pressure 6 psi, solvent flow rate of 70 µL/min, nozzle head velocity of 110 cm/min and 2 mm track spacing. The analysis was performed on a MALDI Fourier-transform ion cyclotron resonance (FTICR) mass spectrometer (solariX 7T-2ω, Bruker Daltonics, Bremen, Germany) equipped with a Smartbeam II 2 kHz laser and operated in positive-ion mode. The experiment was set up in imaging mode at 200 µm lateral resolution firing 100 laser shots per pixel. The average intensity of cyclotide [M+H]^+^ ions per pixel per spot conveyed the relative quantity. Five major cyclotides produced by the *V. uliginosa* suspensions: [M+H]^+^ = 3098.43; 3136.38; 3123.43; 3112.38, 3155.42 were taken into consideration. In a previous work, these peptides were denoted 3097 (an unknown sequence, named according to its monoisotopic molecular weight), cyO2, cyO13, vibe 1 and cyO3, respectively [19]. All spectra were normalized against the root mean square (RMS). The relative quantitative analysis was performed using the msiQuant software [40]. The average relative quantities of particular cyclotides (average intensity per pixel in MALDI-MSI) from the treated and control cultures were compared using a one-way ANOVA with a *t*-test and considered significantly different if *p* < 0.05.

### 4.4. Cyclotide MS/MS Sequencing

The cyclotide denoted in the previous work by its monoisotopic molecular mass—3097 [19]—was identified with its amino acid sequence in the current work. The peptide was extracted, purified, and then sequenced in accordance with well-established protocols [19,22]. Briefly, the leftover biomass from cell cultures was pooled and freeze-dried. The material was extracted with 30% ACN, 0.05% TFA in water and then purified using RP-HPLC. The peptide was then reduced and alkylated using iodocetamide. The reduced and alkylated peptides were purified by size exclusion chromatography (Sephadex G-25 based PD 10 column, Cytiva) and cleaved with Glu-C endoprotease. The MS/MS fragmented peptides resulting from enzymatic cleavage were collected using UPLC-QToF nanospray MS (Waters nanoAcquity, 75 µm × 250 mm 1.7 µm BEH130 C18 column, Waters QToF Xevo). The MS/MS fragmentation spectrum was processed using MaxEnt 3 and the -b and -y ions were assigned using the Peptide Sequencing module in the MassLynx software (Waters). The peptide sequence was searched for in Cybase (http://www.cybase.org.au/index.php accessed on 4 February 2022) and Uniprot (https://www.uniprot.org/ accessed on 4 February 2022) and the absence of any 100% hit proved that the peptide has a novel sequence. The peptide was named using an established nomenclature [41].

## Data Availability

Not applicable.

## Supplementary Materials

The following supporting information can be downloaded at: https://www.mdpi.com/article/10.3390/plants11141876/s1, Figure S1: MSMS de novo sequencing of the peptide fragment resulting from trypsin cleavage of alkylated viul M cyclotide; Figure S2: MSMS de novo sequencing of the peptide fragment resulting from Glu-C (a) or Clu-C, and subsequently trypsin (b,c) cleavage of alkylated vibe 1 cyclotide.
