# Supplementary material for: The Influence of Plant Stress Hormones and Biotic Elicitors on Cyclotide Production in Viola uliginosa Cell Suspension Cultures
Source: Plants (Basel). 2022 Jul 19;11(14):1876. doi: 10.3390/plants11141876 (PMC9324686; doi:10.3390/plants11141876)

Figure S1. MSMS de novo sequencing of the peptide fragment resulting from trypsin cleavage of alkylated viul M cyclotide

Observed MW: 2671.1421

Predicted sequences:

Sequence	Calculated MW	Delta
DGIPCGESCVFIPCLTAAIGCSCK	2671.1575	0.02

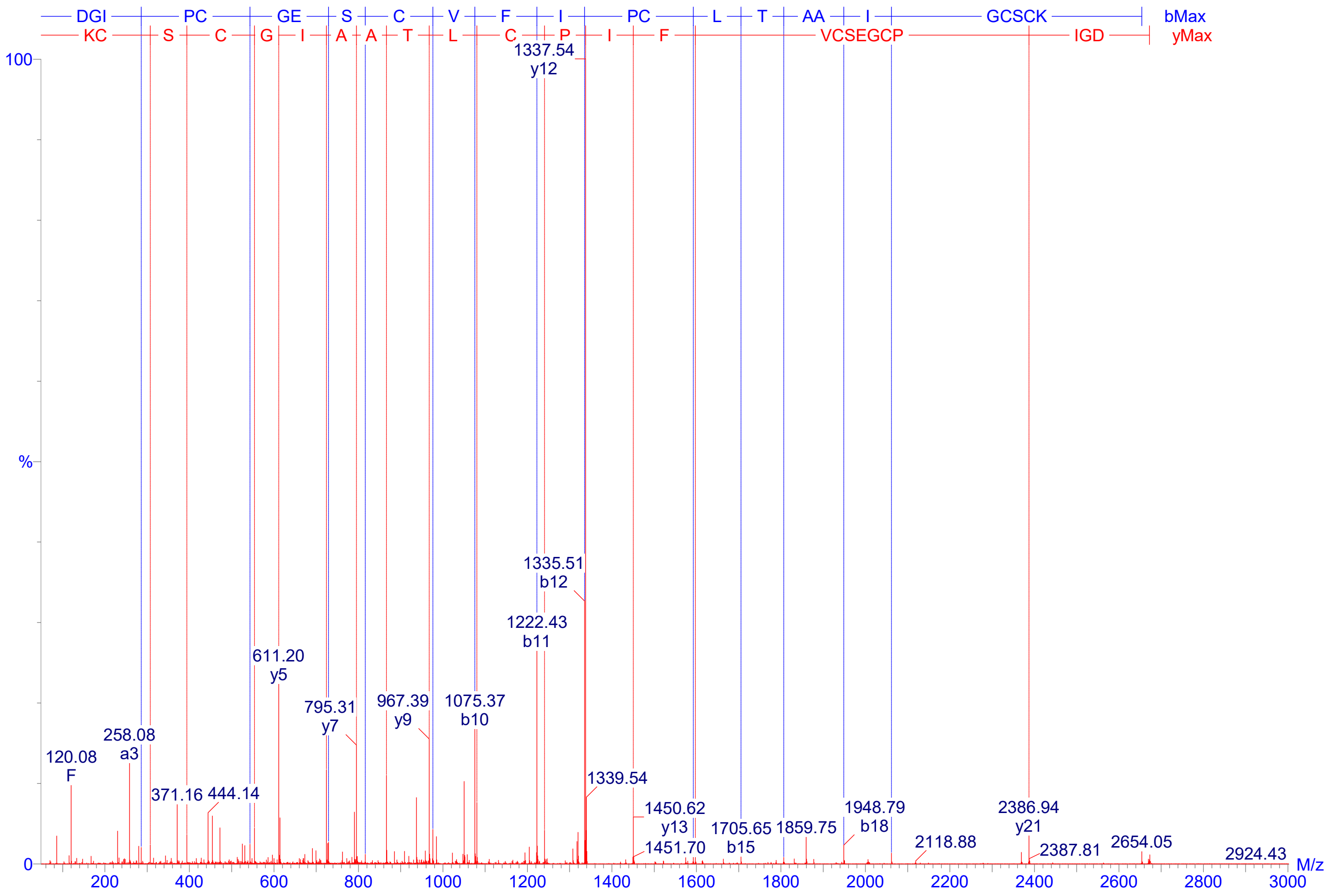

Supplement: Supplementary file 1 [file plants-11-01876-s001.zip › Figure S1.pdf]
