# Supplementary material for: The Influence of Plant Stress Hormones and Biotic Elicitors on Cyclotide Production in Viola uliginosa Cell Suspension Cultures
Source: Plants (Basel). 2022 Jul 19;11(14):1876. doi: 10.3390/plants11141876 (PMC9324686; doi:10.3390/plants11141876)

Figure S2. MSMS de novo sequencing of the peptide fragment resulting from Glu-C (a) or Clu-C and subsequently trypsin (b, c) cleavage of alkylated vibe 1 cyclotide

Observed MW: 3477.5349

(a)

Predicted sequences:

Sequence	Calculated MW	Delta
SCVWIPCITSAIGCSCSSKVCYRNGIPCGE	3477.5068	-0.03

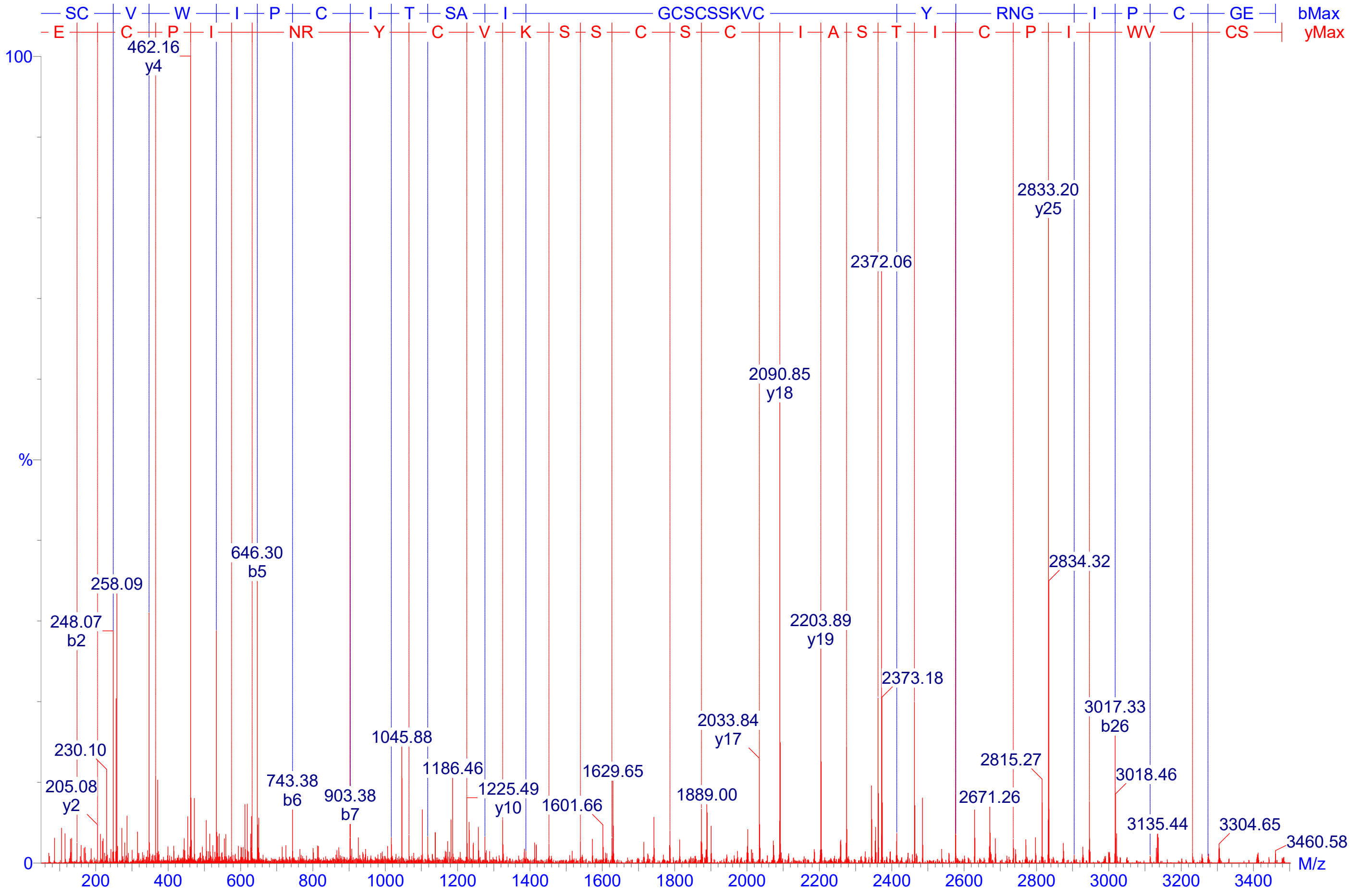

Observed MW: 746.2892

(b)

Predicted sequences:

Sequence	Rank	Calculated MW	Delta
DGIPCGE*	1	746.2905	0.00

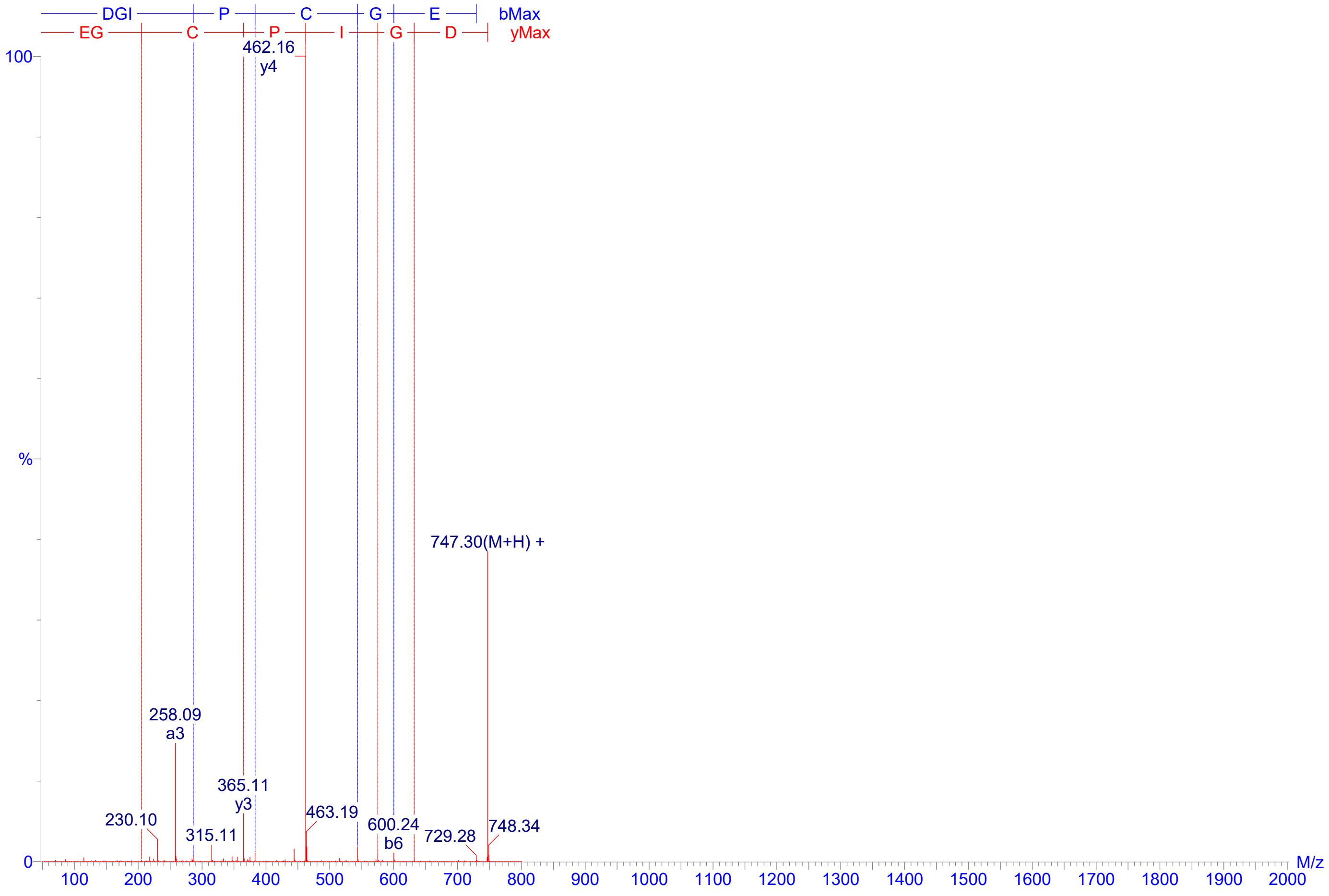

*The peptide was deaminated in the process of alylation therefore the change from N in the native peptide to D in the fragment

(c)

Observed MW: 2171.9341

Predicted sequences:

Sequence	Calculated MW	Delta
SCVWIPCITSAIGCSCSSK	2171.9473	0.01

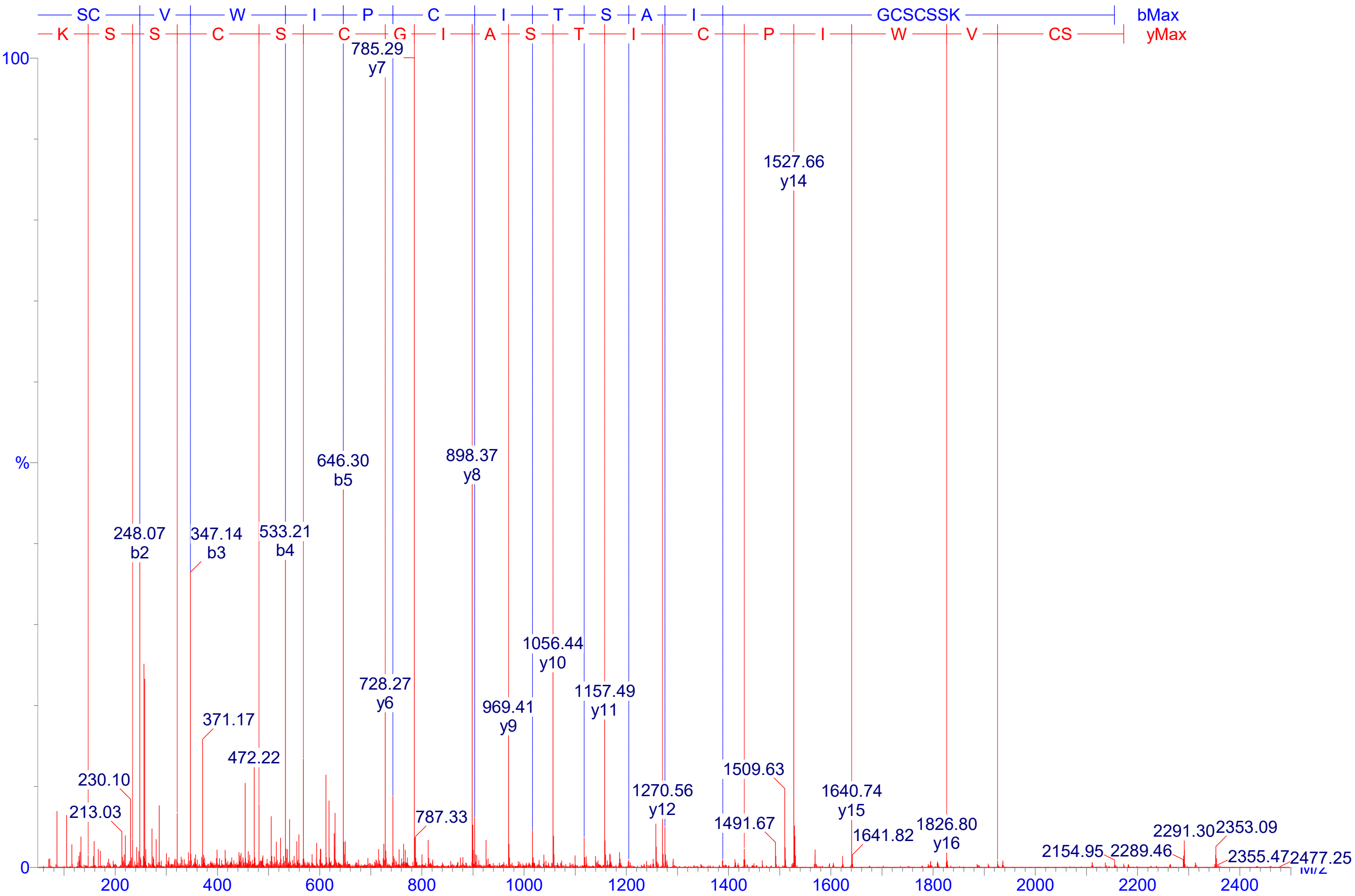

Supplement: Supplementary file 1 [file plants-11-01876-s001.zip › Figure S2.pdf]
